# Use of next‐generation sequencing and candidate gene analysis to identify underlying defects in patients with inherited platelet function disorders

**DOI:** 10.1111/jth.12836

**Published:** 2015-01-27

**Authors:** V. C. Leo, N. V. Morgan, D. Bem, M. L. Jones, G. C. Lowe, M. Lordkipanidzé, S. Drake, M. A. Simpson, P. Gissen, A. Mumford, S. P. Watson, M. E. Daly, the UK GAPP Study Group

**Affiliations:** ^1^ Department of Cardiovascular ScienceUniversity of Sheffield Medical SchoolUniversity of SheffieldSheffieldUK; ^2^ Centre for Cardiovascular SciencesSchool of Clinical and Experimental MedicineCollege of Medical and Dental SciencesUniversity of BirminghamBirminghamUK; ^3^ Schools of Physiology and Pharmacology and Cellular and Molecular MedicineUniversity of BristolBristolUK; ^4^ Division of Genetics and Molecular MedicineKing's College London School of MedicineGuy's HospitalLondonUK; ^5^ Department of Genes, Development and DiseaseInstitute of Child HealthUniversity College LondonLondonUK

**Keywords:** bioinformatics, bleeding, blood platelet disorders, high‐throughput DNA sequencing, platelets

## Abstract

**Background:**

Inherited platelet function disorders (PFDs) are heterogeneous, and identification of the underlying genetic defects is difficult when based solely on phenotypic and clinical features of the patient.

**Objective:**

To analyze 329 genes regulating platelet function, number, and size in order to identify candidate gene defects in patients with PFDs.

**Patients/methods:**

Targeted analysis of candidate PFD genes was undertaken after next‐generation sequencing of exomic DNA from 18 unrelated index cases with PFDs who were recruited into the UK Genotyping and Phenotyping of Platelets (GAPP) study and diagnosed with platelet abnormalities affecting either Gi signaling (*n* = 12) or secretion (*n* = 6). The potential pathogenicity of candidate gene defects was assessed using computational predictive algorithms.

**Results:**

Analysis of the 329 candidate PFD genes identified 63 candidate defects, affecting 40 genes, among index cases with Gi signaling abnormalities, while 53 defects, within 49 genes, were identified among patients with secretion abnormalities. Homozygous gene defects were more commonly associated with secretion abnormalities. Functional annotation analysis identified distinct gene clusters in the two patient subgroups. Thirteen genes with significant annotation enrichment for ‘intracellular signaling’ harbored 16 of the candidate gene defects identified in nine index cases with Gi signaling abnormalities. Four gene clusters, representing 14 genes, with significantly associated gene ontology annotations were identified among the cases with secretion abnormalities, the most significant association being with ‘establishment of protein localization.’

**Conclusion:**

Our findings demonstrate the genetic complexity of PFDs and highlight plausible candidate genes for targeted analysis in patients with platelet secretion and Gi signaling abnormalities.

## Introduction

Inherited platelet function disorders (PFDs), associated with normal or reduced platelet counts, are characterized by lifelong episodes of excessive mucocutaneous bleeding and account for a significant proportion of bleeding diatheses 1. Identification of the underlying genetic defects in these disorders is complicated by the differences in clinical expression of the bleeding symptoms in affected individuals, and the redundancy in platelet receptor and signaling pathways, which mean that defects in the different pathways are not easily discriminated at a phenotypic level.

First described in 1962, light transmission aggregometry (LTA) remains the gold standard method for diagnosing PFDs, while assessment of platelet‐dense granule ATP release provides an additional valuable tool for diagnosing platelet secretion disorders 2. Thus, we have shown that many PFDs can be diagnosed using a combination of LTA and ATP secretion analysis and a streamlined panel of six platelet agonists at specified concentrations 3. Furthermore, most PFDs can be assigned to one of three major diagnostic groups having defects in the thromboxane A_2_ pathway, G_i_ receptor signaling, and dense granule secretion, with the latter two groups accounting for almost two‐thirds of those participants who had a defect in platelet aggregation or ATP secretion 3.

LTA and platelet ATP secretion profiling will identify candidate genes for targeted gene analysis in a small number of PFDs. Previous examples of this phenotype‐driven approach have led to the identification of a homozygous *P2RY12* defect in a patient whose platelets displayed a profile that was consistent with a P2Y_12_ ADP receptor defect and of heterozygous *TBXA2R* defects disrupting function and trafficking of the thromboxane A_2_ receptor in two patients whose platelets demonstrated defects in arachidonic acid metabolism and reduced responses to the thromboxane analogue U46619 4, 5. However, in most cases, identification of the causative gene based solely on the clinical and laboratory phenotype is rarely achieved in PFDs due to the heterogeneity and complexity of these disorders.

Next‐generation sequencing (NGS) technologies that permit the simultaneous analysis of large numbers of genes have facilitated the identification of gene defects in patients with PFDs, where the underlying genetic defect was previously unknown 6, 7, 8, 9, 10, 11, 12, 13. Through the UK Genotyping and Phenotyping of Platelets (UK‐GAPP) study 14, we are combining the power of NGS with targeted analysis of genes that have previously been associated with PFDs in humans or are known or predicted to encode proteins that mediate platelet function, formation, and morphology. This approach has allowed us to identify a novel *HPS4* defect in a patient with Hermansky–Pudlak syndrome 9 and more recently revealed a high incidence of alterations affecting *FLI1* and *RUNX1* in patients presenting with mild bleeding symptoms characterized by defects in platelet‐dense granule secretion 13.

We now describe the results of a targeted analysis of 329 platelet genes, which are known or predicted to have a role in regulating platelet function, size, and number, in 18 unrelated index patients diagnosed with PFDs and recruited to the UK‐GAPP study and shown to have defects in either G_i_ receptor signaling or dense granule secretion.

## Subjects and methods

### Subjects and platelet phenotyping

Index cases from 18 families, recruited through UK Comprehensive Care Haemophilia Centres and enrolled in the UK‐GAPP study between August 2006 and August 2012, were investigated (ISRCTN 77951167). Where available, affected relatives were also investigated. All participants had abnormal bleeding symptoms compatible with a PFD (spontaneous mucocutaneous bleeding or abnormal bleeding following trauma or invasive procedures) and satisfied the criteria for inclusion in the study described earlier, including having coagulation factor levels within the local laboratory reference ranges and no clinical evidence of acquired platelet dysfunction 3. Platelet function testing at the referring centers had previously excluded the possibility of Glanzmann's thrombasthenia, Bernard–Soulier syndrome, or Hermansky–Pudlak syndrome. The study was approved by the National Research Ethics Service Committee West Midlands–Edgbaston (REC reference: 06/MRE07/36), and participants gave written informed consent in accordance with the Declaration of Helsinki. Blood from participants and healthy volunteer subjects was sampled into 3.1% sodium citrate in evacuated tubes (S‐Monovette^®^ 0.106 mol L^–L^; Sarstedt, Leicester, UK) and platelet‐rich plasma was prepared as described previously 3. Platelet aggregation in response to a panel of agonists at different concentrations and ATP secretion were assessed using a dual‐channel Chronolog lumiaggregometer (Model 460 VS; Chronolog, Havertown, PA, USA), as previously described 3. Platelet phenotyping was undertaken for each participant in parallel with a healthy volunteer, and results were compared with the control reference ranges for platelet aggregation and secretion previously established by our group 3, 4.

### Genetic analysis

Genomic DNA was isolated from peripheral blood using the Puregene DNA extraction kit (Qiagen, Manchester, UK) and, after enrichment of coding regions and intron/exon boundaries (10 bp flanking the exons) using the Agilent SureSelect All Exon 50 Mb kit (Agilent Technologies, Wokingham, UK), DNA sequencing was undertaken on the HiSeq 2000 from Illumina (Little Chesterford, UK). Sequence reads were aligned to the reference genome (hg19) using Novoalign (Novocraft Technologies, Sdn Bhd, Malaysia). Duplicate reads and reads that mapped to multiple locations in the exome were excluded from further analysis. Depth of sequence coverage was calculated using custom scripts and the BedTools package 15, and those with a sequence coverage below four were excluded. Single nucleotide variations (SNVs) and small insertions/deletions (indels) were identified and filtered for quality using the SAM tools 16 package and in‐house software tools. Variants were annotated with respect to genes and transcripts using the ANNOVAR tool 17. Further analysis was targeted at variants within a panel of 329 candidate PFD genes, which represents an extension of a previously published list of candidate genes 9 (Data S1). The original list consisted of 216 genes previously associated with PFDs in humans, orthologs of genes linked to platelet dysfunction in selected animal models, and genes that encoded recognized mediators of platelet activation that had not been previously associated with PFDs 9. For this study, the panel was extended to include genes that have been more recently identified as encoding proteins with a role in platelet function, as well as genes encoding proteins involved in the regulation of platelet formation and morphology. Variants within these genes were filtered for novelty by comparison to dbSNP129 and dbSNP132 (EVS; (http://evs.gs.washington.edu/EVS/), the 1000 Genomes Project, and an ‘in‐house’ database of 600 exomes, and those with a minor allele frequency (MAF) > 0.01, as determined by the 1000 Genome Project or EVS, were excluded (Data S2). Novelty was confirmed by comparison to dbSNP138.

### Identification of candidate gene defects

Candidate gene defects were identified using a combination of bioinformatic tools and, where possible, by targeting those variants that were shared by index cases with their affected family members. The potential effects of nonsynonymous amino acid substitutions and indels on protein function were predicted using Sorting Intolerant From Tolerant (SIFT; http://sift.bii.a-star.edu.sg/), Polymorphism Phenotyping v2 (PolyPhen‐2; http://genetics.bwh.harvard.edu/pph2/), and MutationTaster (http://www.mutationtaster.org/). The potential effects on splicing of those variants predicted to be benign by the three latter tools, and all remaining synonymous and intronic variants, were then predicted using Human Splice Finder (HSF; http://www.umd.be/HSF/), SplicePort (http://spliceport.cbcb.umd.edu/), and Alternative Splice Site Predictor (ASSP; http://wangcomputing.com/assp/index.html). Those variants that were predicted to be detrimental by at least one of these bioinformatic tools were considered to be candidate gene defects (Data S2).

### Functional annotation analysis

The Database for Annotation, Visualization and Integrated Discovery (DAVID) (http://david.abcc.ncifcrf.gov) was used to perform functional annotation analysis on those genes identified as having candidate defects. Using the Ensembl gene ID for each gene, a functional annotation chart was generated by selecting the GOTERM_BP_FAT annotation category in DAVID, which allows enrichment analysis to highlight the most relevant Gene Ontology (GO) terms associated with a given gene list. The significance of gene enrichment in annotation terms was measured using the Expression Analysis Systematic Explorer (EASE) score, which is a modified Fisher Exact *P*‐value, with application of the Benjamini correction for multiple comparisons, as applied in DAVID, with the threshold set at 0.05.

## Results

### Characteristics of the participants studied

A subgroup of 18 index cases (F1.1 to F18.1; 13 females and five males), who ranged in age from 6 to 82 years, and were recruited into the UK GAPP study 3, 18 on the basis of abnormal bleeding symptoms and a suspected inherited platelet disorder, were investigated. All index cases had been diagnosed as having PFDs on lumiaggregometry as described earlier 3. Twelve of the index cases (F1.1 to F12.1) were diagnosed as having G_i_‐like abnormalities characterized by transient platelet aggregation in response to 10 μmol ^–L^ ADP and the absence of a secondary wave of aggregation in response to 10 μmol ^–L^ epinephrine. The other six index cases (F13.1 to F18.1) were classified as having dense granule secretion defects, which were characterized by a reduction in ATP secreted in response to protease‐activated receptor–1 specific peptide (SFLLRN; 100 μmol L^–L^ ) to a level below the 5th percentile for the response in healthy volunteers 3. In addition to the index cases, we studied four individuals who were first‐degree affected relatives of each of the index cases (F1.1 to F4.1). These individuals, denoted F1.2 to F4.2, also had G_i_ signaling abnormalities.

### Exome sequencing and target gene analysis

Exome sequencing and targeted analysis of selected platelet genes were undertaken for all participants (18 index cases, F1.1 to F18.1, and four affected relatives, F1.2 to F4.2). After calculation of the depth of sequence coverage, variants with a coverage of less than four were removed from any further analysis. The average read depth for the remaining variants across all samples was 106.4. Alignment of exome sequence data to the human genome identified ~25 000 variants in the exome of each participant (Data S3). Restriction of the analysis to 329 candidate PFD genes, based largely on a previously described list 9, reduced the number of variants to < 350 per participant. Filtering of the data for novelty, and removal of those variants having MAFs > 0.01, reduced the number to < 30 per participant. After removal of those variants that were predicted to be benign using all bioinformatic tools described in Methods, a median of 4.5 (range 2–9) candidate gene defects were identified in those participants with G_i_ signaling abnormalities and 7.5 (range 3–11) in those participants with secretion defects (Data S3). For those four index cases where an affected family member was also investigated, the number of potentially deleterious candidate gene defects was reduced by ~50% after filtering for those variants that were shared between affected individuals.

### Identification of candidate gene defects in patients with G_i_ signaling abnormalities

A total of 63 candidate gene defects, within 40 genes, were identified among the 12 index cases with G_i_ signaling abnormalities, of which 70% (44/63) were missense alterations and 17 were previously unreported (Table 1). In addition to the candidate missense alterations, eight indels, one of which was predicted to cause a frame shift, and 11 potential splicing defects were identified. All candidate variations identified in this cohort were inherited as heterozygous defects (Table 1).

**Table 1:** Genotype and nature of candidate gene defects identified in two subgroups of index cases

Variant type	G_i_ signaling abnormality (*n* = 12)				Secretion abnormality (*n* = 6)			
	Hetero	Homo	Total	Novel	Hetero	Homo	Total	Novel
Missense	44	0	44	15	34	2	36	15
Indel/frame shift	1	0	1	0	2	3	5	5
Indel/inframe	7	0	7	2	1	2	3	3
Splicing	11	0	11	0	8	1	9	2
Total	63	0	63	17	45	8	53	25

Indel/frame shift, insertion or deletion predicted to cause a frame shift; indel/inframe, insertion or deletion that does not disrupt the reading frame; hetero, heterozygous variant; homo, homozygous variant.

To prioritize the candidate gene defects, functional annotation analysis was undertaken using the DAVID tool 19, which, for a given list of genes, clusters gene products in terms of their associated biological processes, cellular components, and molecular functions, by identifying those significantly associated GO annotations. Functional annotation analysis of the 40 genes that were identified as having the 63 candidate gene defects among the index cases with G_i_ signaling abnormalities, revealed a single cluster, comprising 13 genes, which showed significant annotation enrichment for ‘intracellular signaling’ (*P* = 0.0038). This cluster harbored 16 of the candidate gene defects that were identified in nine of the 12 index cases with G_i_ signaling abnormalities (Tables 2 and 3).

**Table 2:** Functional annotation analysis of genes harboring candidate defects in two subgroups of index cases

Annotation term	*P* value^a^	No. of genes
G_i_ signaling abnormality		
Intracellular signaling	3.8 × 10^−3^	13
Secretion abnormality		
Establishment of protein localization	3.2 × 10^−4^	13
Protein transport	5.9 × 10^−4^	13
Protein localization	4.1 × 10^−4^	13
Vesicle‐mediated transport	8.3 × 10^−4^	11

a Terms are sorted based on the Benjamini‐corrected Modified Fisher Exact *P*‐value as reported in DAVID (http://david.abcc.ncifcrf.gov). *P *< 0.05 denotes significance.

**Table 3:** Genes harboring defects among index cases with abnormalities in G_i_ signaling and showing enriched annotation by functional annotation analysis

Patient ID	Gene	Nucleotide change	Amino acid change	Zygosity	Effect	dbSNP ID	1000 genomes MAF	EVS MAF
F3.1	*P2RY12*	c.365G>A	p.R122H	Heterozygous	Missense	Novel		
F12.1		c.772C>A	p.P258T	Heterozygous	Missense	rs202099742	0.000077	No data
F7.1	*PLCB3*	c.130C>T	p.R44C	Heterozygous	Missense	Novel		
F1.1	*UNC13A*	c.1032_1034delGGA	p.E344del	Heterozygous	Inframe deletion	Novel		
F8.1		c.739G>A	p.E247K	Heterozygous	Missense	rs200805380	0.00024	No data
F8.1	*VAV2*	c.1998C>G	p.I666M	Heterozygous	Missense	rs144269361	0.0009	No data
F9.1	*RGS19*	c.650C>T	p.A217V	Heterozygous	Missense	Novel		
F2.1	*TLR2*	c.2186A>G	p.N729S	Heterozygous	Missense	rs61735278	0.001	0.0005
F3.1	*PTGIR*	c.44T>C	p.V15A	Heterozygous	Splice defect	rs200213497	0.002035	No data
F5.1	*PLA2G4C*	c.410C>T	p.A137V	Heterozygous	Missense	rs11564532	0.001615	0.0009
F11.1		c.454G>T	p.V152F	Heterozygous	Missense	rs11564534	0.009918	0.0046
F3.1	*ARHGEF12*	c.989A>G	p.H330R	Heterozygous	Splice defect	rs187048571	0.000835	0.0011
F1.1	*PTPRC*	c.367G>C	p.D123H	Heterozygous	Splice defect	rs41269905	0.0085	0.01399
F8.1	*ADCY6*	c.358C>T	p.R120C	Heterozygous	Missense	rs55770045	0.01261	0.0109
F5.1	*PDZD3*	c.291G>A	p.A97=	Heterozygous	Splice defect	rs150917049	0.000385	No data
F7.1	*PRKD1*	c.1181G>A	p.R394K	Heterozygous	Splice defect	Novel		

MAF, minor allele frequency; EVS, exome variant server.

### Identification of candidate gene defects in patients with dense granule secretion abnormalities

Fifty‐three candidate defects, within 49 genes, were identified among the patients with dense granule secretion defects, approximately half (25/53) of which had not been reported previously. Missense alterations accounted for 68% (36/53) of the candidate defects identified. Eight indels and nine potential splicing defects were also identified. Five of the indels were predicted to result in frame shifts (Table 1). Interestingly, homozygous candidate gene defects were more commonly associated with secretion abnormalities. Thus, eight candidate gene defects (two missense, five indels, and one splicing defect), affecting eight different genes, were inherited as homozygous alterations in four of the patients with dense granule secretion defects (Table 1). Three of these patients harbored more than one homozygous defect, and each of the four patients had also inherited between seven and 11 additional heterozygous candidate defects in the panel of 329 genes.

Functional annotation analysis was carried out for the 49 genes that harbored the 53 candidate gene defects identified among the index cases with secretion abnormalities. This revealed four clusters of between 11 and 13 genes that had significantly associated GO annotations (Tables 2 and 4). There was considerable overlap in the genes present in each cluster, with a total of 14 genes being represented by all four clusters, with the most significant enrichment being for ‘establishment of protein localization’ (*P* = 0.00032). Of the eight genes bearing candidate homozygous defects, only one, *HOOK3*, remained after prioritization of the candidate genes using functional annotation analysis. There was no overlap between the 13 genes that showed significant annotation enrichment for ‘intracellular signaling’ among patients with G_i_ signaling abnormalities and the 14 genes represented by four clusters identified by functional analysis in the patients with dense granule secretion abnormalities.

**Table 4:** Genes harboring defects among index cases with platelet secretion abnormalities and showing enriched annotation by functional annotation analysis

Patient ID	Gene	Nucleotide change	Amino acid change	Zygosity	Effect	dbSNP ID	1000 genomes MAF	EVS MAF
F14.1	*VAV1*	c.630T>C	p.T210=	Heterozygous	Splicing defect	Novel		
F18.1	*STX7*	c.572A>G	p.D191G	Heterozygous	Missense	rs144864017	0.000231	No data
F18.1	*STX2*	c.94T>G	p.F32V	Heterozygous	Missense	rs137928907	0.018761	0.0089
F18.1	*STXBP4*	c.287+7A>G	N/A	Heterozygous	Splicing defect	rs181690254	0.003921	0.0023
F15.1	*LYST*	c.176‐1G>A	N/A	Heterozygous	Splicing defect	rs141317482	0.004691	0.0051
F13.1	*STXBP5L*	c.G1135A	p.V379M	Heterozygous	Missense	rs61996323	0.006289	0.006
F16.1	*VPS41*	c.2528G>A	p.R843H	Heterozygous	Missense	rs1059508	0.020145	0.0069
F17.1	*SYTL3*	c.259G>T	p.V87L	Heterozygous	Missense	Novel		
F17.1		c.205G>T	p.V69L	Heterozygous	Missense	N/A	No data	0.0014
F17.1	*VPS4B*	c.1329A>C	p.E443D	Heterozygous	Missense	Novel		
F13.1	*HOOK3*	c.1945‐3dup	N/A	Homozygous	Splicing defect	Novel		
F18.1	*VPS16*	c.515‐10C>T	N/A	Heterozygous	Splicing defect	rs45564738	0.006843	0.0032
F18.1	*SCFD1*	c.1021A>G	p.T341A	Heterozygous	Missense	rs1754285	0.01464	0.0073
F13.1	*STXBP1*	c.38‐3T>C	N/A	Heterozygous	Splicing defect	rs138763389	0.013994	0.0074
F15.1		c.325+8C>T	N/A	Heterozygous	Splicing defect	rs117372398	0.003383	0.0014
F17.1	*STXBP2*	c.828‐4C>T	N/A	Heterozygous	Splicing defect	rs151257815	0.013686	0.0078

MAF, minor allele frequency; EVS, exome variant server.

## Discussion

In this study, we have investigated the use of exome sequencing and targeted gene analysis to identify candidate gene defects in PFDs by studying 12 index cases diagnosed with G_i_ signaling abnormalities and six index cases diagnosed with platelet secretion defects. Inherent in our approach was our prediction that candidate defects occur in genes encoding platelet proteins that function in the same or related pathways among patients with similar phenotypes. For example, a defect in G_i_ signaling may be explained by one or more alterations in the genes encoding the G_i_‐coupled platelet receptors and the proteins participating in their downstream signaling pathways. Likewise, a reduction in ATP secretion could be due to one or more defects in genes regulating granule formulation and/or exocytosis.

Comparison of the exome sequence data with the human genome sequence identified ~350 variants in the panel of 329 candidate platelet function defect genes for each index case. A combination of strategies was used to exclude those variants that were unlikely to contribute to the phenotype observed in the index cases and enrich the data for potentially causative gene defects. The data were first filtered by excluding common variants listed on public and in‐house databases and restricting the analysis to unique or rare variants (MAF < 0.01), which were predicted to be deleterious using a selection of bioinformatic tools. This identified a number of plausible candidate gene defects with a median of 4.5 potentially deleterious defects remaining in the index cases with G_i_ signaling abnormalities and 7.5 in those with secretion defects following use of these strategies to filter the exome sequence data, which could be reduced by ~50% by considering, where possible, only those variants that were shared with an affected relative. Because unaffected family members were not recruited to the study, it was not possible to achieve a further reduction in the number of plausible candidate gene defects by excluding those candidate variants that were also present in DNA from unaffected relatives. Nevertheless, these findings emphasize the heterogeneous nature of inherited PFDs, and the likely contribution of multiple genetic defects, which also interact with acquired factors, to determine the overall expression of a bleeding tendency. Interestingly, homozygous candidate gene defects were more common among those index cases diagnosed with secretion abnormalities than among those with G_i_ signaling defects (Fisher's Exact test *P* = 0.0014), suggesting that abnormalities in platelet secretion may be more likely to be inherited as autosomal recessive traits and to be due to severe or complete loss of function, rather than haploinsufficiency, of specific platelet proteins. Additionally, some of the heterozygous candidate gene defects that have been identified occur in genes that are usually associated with autosomal recessive traits. For example, defects in *LYST* are associated with the rare autosomal recessively inherited Chediak–Higashi syndrome, while platelet P2Y_12_ receptor deficiency is usually due to recessive inheritance of *P2RY12* defects. Our findings suggest that heterozygous defects in these genes could affect platelet function as dominant traits and contribute to the expression of a mild bleeding tendency. Indeed, it is plausible that a heterozygous defect in one of these genes could, in combination with a heterozygous defect in one or more related genes, such as genes encoding proteins that function in the same signaling or trafficking pathway, contribute to the overall bleeding risk. In support of this, we have previously described two heterozygous *P2RY12* defects in patients with type 1 von Willebrand disease and shown that these are likely to contribute to the bleeding tendency in the families with the defects providing further evidence for locus heterogeneity in type 1 von Willebrand disease 20, 21. Similarly, our findings in this study emphasize the heterogeneity and likely polygenic inheritance of mild platelet bleeding disorders.

Defects were identified in 40 of the 329 platelet genes analyzed among the patients with the G_i_ signaling abnormalities and in 49 genes among the patients with the dense granule secretion defects. Ten genes were identified as bearing defects in both patient subgroups. Given the different phenotypes, the defects in these 10 genes are unlikely to explain the platelet abnormalities in the patients. Furthermore, the overlap in gene sets between the two patient subgroups indicates that further prioritization of the candidate gene defects would be desirable before undertaking further studies to investigate their potential contribution to the platelet abnormalities in the patients. A handful of the candidate defects were present in genes that had previously been associated with platelet bleeding disorders, including *P2RY12*,* VPS33B*,* GATA1*,* ANKRD26*,* HPS1*,* VWF,* and *LYST* 22. However, the majority of candidate defects were present in genes that have not previously been associated with platelet bleeding disorders in humans.

We also investigated the utility of functional annotation analysis as a tool for prioritizing candidate gene defects for follow‐up studies of their potential role in PFDs that are characterized by defects in G_i_ signaling and secretion. This approach identified a single cluster of 13 genes that showed significant annotation enrichment for intracellular signaling, and harbored defects in nine of the 12 patients whose platelets displayed G_i_ signaling abnormalities, characterized by reduced aggregation in response to ADP and epinephrine. Interestingly, this cluster includes *P2RY12* encoding the P2Y_12_ ADP receptor, which is an essential component of the G_i_‐linked receptor pathway that is necessary for full platelet activation 23. Two candidate defects predicting amino acid substitutions in *P2RY12* were identified in two patients. One of these, predicting a p.P258T substitution in the third extracellular loop of the receptor, was previously identified as a heterozygous defect in a patient with abnormal bleeding, whose platelets failed to aggregate in response to low concentrations of ADP 24. The second predicted a p.R122H substitution in the highly conserved DRY motif of P2Y_12_ which is thought to play a role in regulating conformational states of the receptor. This mutation has not been described previously. However, our group has recently described a patient with a homozygous mutation predicting substitution of the same arginine residue by cysteine. The recombinant R122C variant showed reduced cell surface expression in platelets and agonist‐independent internalization when expressed in cell lines 25. Further work will be required to determine whether the R122H variant behaves similarly. Defects in *ADCY6* and *PTGIR*, which encode adenyl cyclase and the prostacyclin receptors, respectively, are also included in this cluster. These could potentially cause an increase in intracellular cAMP and inhibition of platelet aggregation, opposing the effect of G_i_ signaling and appearing as impaired G_i_‐type responses on platelet function testing 26.

All 14 genes highlighted by functional annotation analysis of the genes bearing defects among the index cases with secretion abnormalities encode proteins involved in protein localization or transport, and all six index cases possessed a mutation in at least one of these genes. Of particular note, six genes in this group encode members of the syntaxin family of proteins, known for their role in SNARE‐complex activity 27, mediating vesicle formation, transport, and secretion from the cell. Thus, 67% of the index cases diagnosed with a dense granule secretion defect had alterations in one of the syntaxin genes, further supporting a role for the SNARE‐complex in platelet‐dense granule biogenesis and secretion. Although homozygous defects were found to be more common among the patients with the dense granule secretion disorders, only one of the eight genes that were shown to bear candidate homozygous defects remained after functional annotation analysis of the data. This was a homozygous splice site defect in *HOOK 3*, which encodes a microtubule tethering protein that is believed to have a role in vesicle trafficking 28, and the patient with this defect (F13.1) was also heterozygous for a splicing defect in *STXBP1* and a missense mutation in *STXBP5L*. Further work is required to determine the relative contribution, if any, of these defects to the bleeding tendency in this patient.

Importantly, functional annotation analysis has identified distinctly different sets of genes associated with G_i_ signaling and secretion abnormalities and has allowed segregation of the two groups of patients. This finding highlights the value of platelet phenotyping not only in allowing assignment of PFDs into diagnostic subgroups but also in directing downstream studies leading to the identification of the underlying genetic defects. Further studies will be required to determine which, if any, of the candidate defects identified in this study explain the different bleeding disorders, although the functional annotation analysis has highlighted plausible candidates. However, it remains likely that the bleeding diatheses experienced by the index cases and affected family members are explained by the specific combination of candidate platelet gene defects inherited and that the expression of the bleeding tendency will, in turn, be affected by an interaction with other nongenetic factors, such as surgery, to determine the overall risk of bleeding.

While exome sequencing and targeted platelet gene analysis have allowed us to identify and prioritize candidate gene defects that could underlie or contribute to the platelet function defects observed in the index cases studied, our approach does have limitations. It is possible that other causative gene defects have been overlooked in our panel of platelet genes as a result of the strategies used to prioritize the candidate gene defects for further follow‐up studies. Causative gene defects in the non‐coding regions of the targeted platelet genes, which are not included in the exome, or indeed in other genes that are not in our panel of platelet genes, would also be overlooked by our approach. The potential for other genetic mechanisms, such as copy number variation, large indels, or structural genomic variants, to contribute to the underlying defects also cannot be discounted. Although further studies are required to confirm the pathogenicity of the candidate defects identified in this study, our approach has highlighted several plausible candidate genes for targeted analysis in patients with abnormalities of platelet secretion and G_i_ signaling abnormalities.

## Addendum

V. C. Leo and M. E. Daly wrote the manuscript, which was read and commented on by all authors. V. C. Leo, N. V. Morgan, D. Bem, M. L. Jones, G. C. Lowe, M. Lordkipanidzé, S. Drake, and M. A. Simpson contributed to the data and laboratory analyses. G. C. Lowe, M. Lordkipanidzé, A. Mumford, and P. Gissen recruited patients and contributed clinical data to the study. M. E. Daly and S. P. Watson coordinated the study.

## Disclosure of Conflict of Interests

S. Drake reports grants from University of Birmingham during the conduct of the study. All other authors state that they have no conflicts of interest.

## Supporting information


**Data S1.** List of 329 candidate platelet function defect genes.

**Data S2.** Exome sequencing and targeted genetic analysis pipeline.

**Data S3.** Median number of variants identified during sequence analysis for two subgroups of index cases.
