# Supplementary figures and images for: Use of next‐generation sequencing and candidate gene analysis to identify underlying defects in patients with inherited platelet function disorders
Source: J Thromb Haemost. 2015 Jan 27;13(4):643–50. doi: 10.1111/jth.12836 (PMC4383639; doi:10.1111/jth.12836)

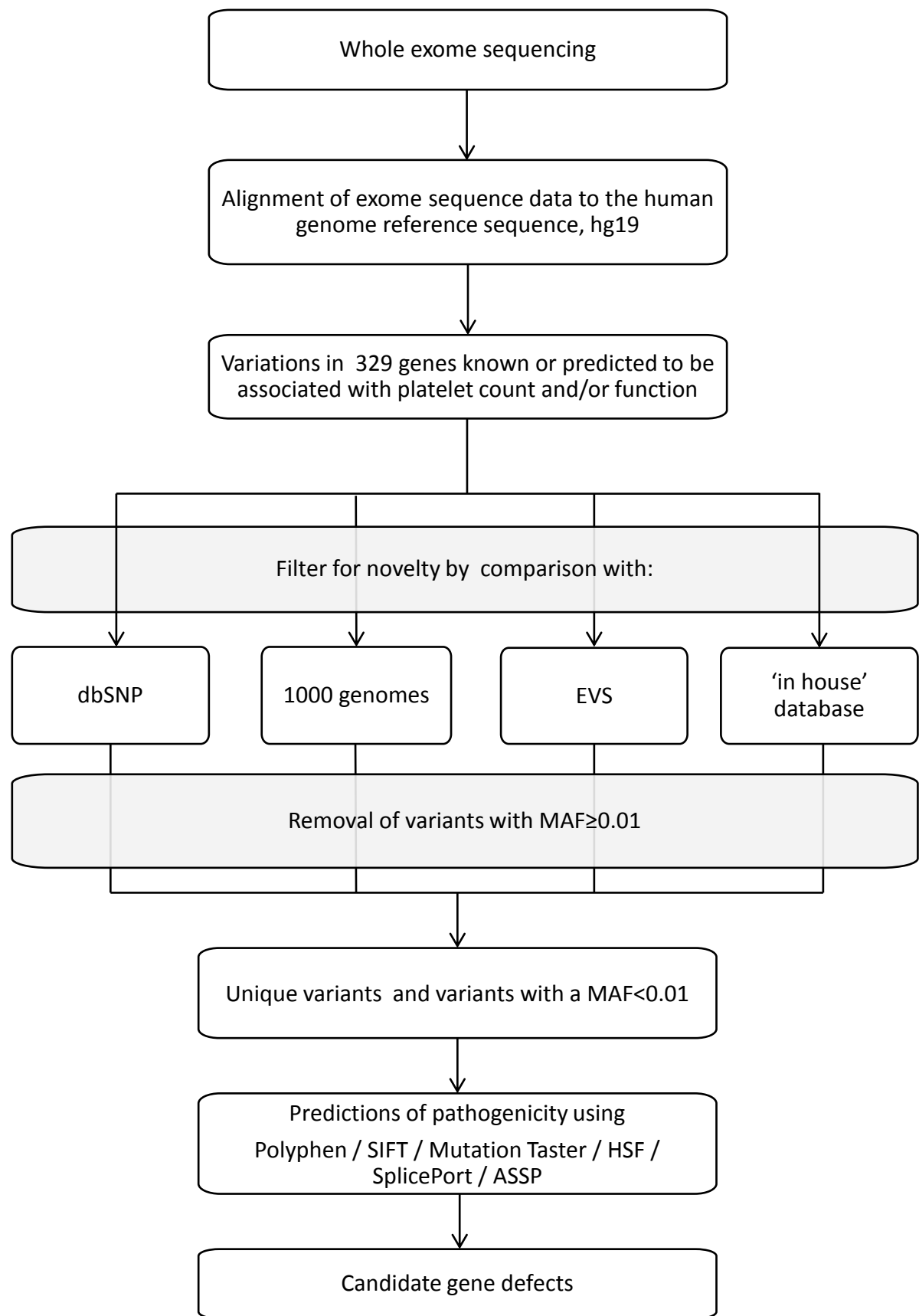

Supplement: Supplementary file 2 — Data S2. Exome sequencing and targeted genetic analysis pipeline. [file jth0013-0643-sd2.pdf]
